# Identification of Differentially Expressed Genes after Endurance Runs in Karbadian Horses to Determine Candidates for Stress Indicators and Performance Capability

**DOI:** 10.3390/genes14111982

**Published:** 2023-10-24

**Authors:** Monika Reißmann, Abirami Rajavel, Zaur A. Kokov, Armin O. Schmitt

**Affiliations:** 1 Thaer-Institute of Agricultural and Horticultural Sciences, Humboldt-Universität zu Berlin, Unter den Linden 6, 10099 Berlin, Germany; monika.reissmann@agrar.hu-berlin.de; 2 Breeding Informatics Group, Department of Animal Sciences, Georg-August University, Margarethe von Wrangell-Weg 7, 37075 Göttingen, Germany; 3 Institute of Physics and Mathematics, Kabardino-Balkarian State University, Chernyshevsky 173, Nalchik 360004, Russia; zak@kbsu.ru; 4 Center for Integrated Breeding Research (CiBreed), Georg-August University, Carl-Sprengel-Weg 1, 37075 Göttingen, Germany

**Keywords:** horse, endurance performance, differential gene expression, long-distance run, short-distance run

## Abstract

RNA sequencing makes it possible to uncover genetic mechanisms that underlie certain performance traits. In order to gain a deeper insight into the genetic background and biological processes involved in endurance performance in horses, the changes in the gene expression profiles induced by endurance runs over long (70 km) and short (15 km) distances in the blood of Kabardian horses (*Equus caballus*) were analyzed. For the long-distance runs, we identified 1484 up- and 691 downregulated genes, while after short-distance runs, only 13 up- and 8 downregulated genes (FC > |1.5|; *p* < 0.05) were found. These differentially expressed genes (DEGs) are involved in processes and pathways that are primarily related to stress response (interleukin production, activation of inflammatory system) but also to metabolism (carbohydrate catabolic process, lipid biosynthesis, NADP metabolic process). The most important genes involved in these processes therefore represent good candidates for the monitoring and evaluation of the performance of horses in order to avoid excessive demands when endurance performance is required, like *ACOD1*, *CCL5*, *CD40LG*, *FOS*, *IL1R2*, *IL20RA*, and *IL22RA2*, on the one hand, and, on the other hand, for assessing the suitability of a horse for endurance races, like *GATA2*, *GYG1*, *HIF1A*, *MOGAT1*, *PFKFB3*, *PLIN5*, *SIK1*, and *STBD1*.

## 1. Introduction

To date, numerous genomic polymorphisms have been discovered in horses that are associated with traits relevant to breeding. However, these are primarily mutations that are associated with monogenic phenotypic properties such as hereditary defects [1], coat color [2], or appearance [3]. However, there is a distinct lack of knowledge about the genetic basis of athletic performance, which is the focus of many breeders. Thanks to modern methods of molecular genetics and bioinformatics, initial successes have also been achieved here [4,5,6]. For example, the *myostatin* gene (*MSTN*), which is involved in the formation and development of muscle fibers, has been identified as a strong candidate gene for running ability [7,8]. Several mutations are known to be associated with race distance fitness [9,10] and body composition [11] in Thoroughbreds. Additional candidate genes have been found in Arabians [12] and Quarter Horses [13]. However, the sporting use of horses covers more areas than just racing performance. Good skills in jumping, dressage, Western riding, or mastering different gaits significantly expand the range of desired characteristics. Here, too, initial successes were achieved, which led, for example, to the identification of QTLs for jumping [14] or a mutation in the *DMRT3* gene associated with gait [15].

However, all these genes found are mainly major genes in which individual polymorphisms in protein-coding regions have an above-average influence on the traits of interest. However, most properties are caused by complex metabolic processes in which not only numerous genes with their specific nucleotide sequences are involved, but which can also be modulated by environmental factors. Analyses using cDNA microarrays, RNA-seq, or qPCR are one way to obtain a deeper insight into the underlying biological processes and their genetic basis. By comparing gene expression in different states such as before and after specific exercises [16,17] and in different muscles [18,19], blood vs. muscle [20], or in various breeds [21,22] and hybrids [23], it may be possible to identify the genes involved in the biological context. Further investigations, in which analysis of eQTLs [24], noncoding RNAs [25,26], or changes in the methylation pattern [27,28,29] are in the foreground, deal with possible causes of expression changes, try to uncover alternative splice variants [17,18], selective isoform expression, or protein-/expression-changing SNPs in the genes involved [30,31,32], or focus on the combination of several omics levels [33,34,35] to better understand the relationships between genotype and phenotype as well as the influence of the environment. The final result is to determine the genetic influences on diseases and, conversely, the genetic background for good health, welfare, and performance [36,37,38].

Endurance rides have become increasingly popular in recent years, both for competition and for leisure. Endurance, which is defined as the ability to perform over a longer period of time, plays a major role here. Therefore, this criterion is not only of interest for endurance rides, but for almost all types of use of the horse. However, there is only insufficient knowledge about the biochemical–physiological basis of this characteristic. In addition, it is still unclear how it can be quantified at all, since it is extremely complex and involves the interaction of numerous factors. Studies in this area have shown repeatedly how closely intertwined the various metabolic pathways are, on the one hand, and how environmental factors can have a modeling effect on expression, on the other hand. Bou et al. [39] showed that endurance training leads to alternative splicing and exon-skipping events as well as differentially expressed noncoding RNAs. The modulation of noncoding RNAs, which ultimately causes altered post-transcriptional regulation of gene expression and, thus, metabolic as well as immune adaptation, in response to exercise were shown in various studies [25]. As circulating miRNAs, they correlate with different reactions to endurance stress and are therefore considered to be more accurate biomarkers than serum markers [26,40].

A particularly important characteristic for good endurance horses is the moderate handling of stress. Stress can have an impact on different levels. Important types of stress include oxidative, heat, hypoxic, hormonal, and glucose stress [41]. In addition to adrenal gland hormones, immunological markers react to stress, meaning that their level can be seen as a marker for stress. This has been demonstrated, for example, for various interleukins and TNF-α both at the mRNA expression level [42,43] and at the protein level (serum concentration) [44,45,46]. These studies are important because precise knowledge of stress indicators, such as cytokines, offers an opportunity to correctly assess the training level of horses and thus help not to overload horses in sporting competitions. This addresses welfare issues that play a major role in dealing with horses today.

In our study, RNA sequencing data from seven Kabardian horses, generated before and after runs over two different distances (70 km and 15 km), are used to identify genes involved in endurance and gain an insight into the underlying physiological processes of sustained work. We pursued two goals: (i) Search for genes associated with stress-coping in order to find indicators to monitor health and the level of effort; (ii) Identification of candidate genes that are directly related to performance and can therefore be of interest for breeding and selection of endurance horses.

## 2. Material and Methods

### 2.1. Animals, Endurance Ride Conditions, and Ethics Statement

We studied seven adult Kabardian horses (*Equus caballus*) in endurance riding at two different locations. Three animals participated in an endurance ride over a 70 km distance in the Caucasus (Russia). After 35 km, there was a one-hour break and a vet check (long-distance group). The other four animals took part in a ride in the Bavarian Forest (Germany) covering a 15 km distance without break (short-distance group). The terms long- and short-distance do not refer to the categories used in endurance races, where only runs of 100 km or more are considered long-distance runs and runs of 15 km are not considered endurance rides. We use them to make a brief and clear distinction between our two distance groups. In the Caucasus, the route was in the first stage at a height of 630 to 750 m above sea level with an average speed of 14 km/h and in the second stage at 630 m to 1200 m above sea level with an average of 10 km/h. In the Bavarian Forest, the height was between 450 m and 600 m above sea level with an average speed of 12 km/h. Temperature and humidity were moderate. The endurance rides were practice rides. The field and forest paths were easy to ride on. All horses were well trained and had already completed such distance rides. All experimental procedures used in this study were performed in accordance with the animal welfare laws of both countries and were approved by the respective animal welfare organizations (Russia: No. 1571, Germany: 55.2-1-54-2532-128-1514). The horses were monitored by veterinarians during the rides.

### 2.2. Sample Collection and RNA Sequencing

A total of 10 mL of EDTA blood was taken from the jugular vein of each horse shortly before, T_1_ (15 min), and immediately after the end, T_2_, of the endurance ride. The total RNA from this blood was immediately secured using the LeukoLOOK-KIT Total RNA System from Ambion by Life Technologies (Carlsbad, CA, USA). With this kit, cellular fractionation of the whole blood was carried out immediately after blood collection. The leukocyte population obtained was transported in a stabilizing RNAlater for further processing in the laboratory. After extraction of total RNA according to the manufacturer’s instructions, which also significantly reduced the proportion of globin mRNA (negative effect on downstream expression analyses), the RNA quality was checked using a BioAnalyzer 2100 (Agilent, Santa Clara, CA, USA) with an RNA chip, and storage took place at −80 degrees Celsius until sequencing.

The total RNA sequencing, including the library preparation, was carried out by LGC Genomics (Berlin, Germany) following Illumina’s specifications. The NuGEN Ovation RNA-seq library preparation system (Illumina Inc., San Diego, CA, USA) was used to create the mRNA libraries. The 75 bp paired-end reads were generated with the Illumina NextSeq 500 V2 system (Illumina Inc., San Diego, CA, USA) for all seven animals. Altogether, 28 libraries (7 animals × 2 time points (before and after the run) × 2 directions (forward and reverse)) of RNA sequences comprising 582 million reads were generated. The number of reads in a library ranged from 11.4 million to 28.8 million, with a mean of 18.8 million reads.

### 2.3. Data Processing

We downloaded the horse reference genome Equus caballus version EquCab3.0 (file: Equus_caballus.EquCab3.0.DNA.toplevel.fa.gz) and the gene annotation file (Equus_caballus.EquCab3.0.108.gtf.gz) from the Ensembl ftp-server https://www.ensembl.org/info/data/ftp/ndex.html (accessed on 7 November 2022). The adapter clipped short reads were mapped to the reference genome using STAR 2.7.4a [47], which yielded alignment files (so-called bam files). From these alignments, we extracted the gene counts using htseq-count [48]. The R programming environment was used for general data handling and standard analysis. The Venn diagram was produced with the online tool available under https://bioinformatics.psb.ugent.be/webtools/Venn/ (accessed on 14 November 2022).

### 2.4. Analysis of Differentially Expressed Genes

For the identification of differentially expressed genes, the two groups of animals were considered separately: the three animals (*n* = 3) that completed the long distance (70 km) and the four animals (*n* = 4) that completed the short distance (15 km). We term these groups as the following: “long-distance” and “short-distance” groups.

Differentially expressed genes (DEGs) were identified with the R-package DESeq2 [49] based upon unnormalized count data. The function DESeq of that package is used to calculate the dual logarithm of the fold changes (log2FC). Adjusted *p*-values and false discovery rates (FDR) were calculated with the R-function p.adjust. Genes with an absolute value of the log fold change > 0.58 (i.e., a fold change of over 1.5 or under 0.67), and an adjusted *p*-value < 0.05 were considered as differentially expressed, as was performed in other studies [16,50,51]. Hence, we speak of (statistically significant) upregulation of a gene if its expression was, on average, at least 50% increased after the run (T_2_, also termed “post-run”) with respect to its expression before the run (T_1_, also termed “pre-run”). Likewise, we speak of downregulation of a gene if its expression was, on average, at least 33% decreased after the run with respect to its expression before the run. To easily distinguish up- and downregulated genes, we use, in all tables, a minus sign to indicate downregulated genes while upregulated genes are shown as a positive number, which is in line with the nomenclature used in [52,53].

### 2.5. Functional Enrichment Analysis of Differentially Expressed Genes

Functional enrichment analysis of the identified DEGs was performed with g:Profiler https://biit.cs.ut.ee/gprofiler (accessed 30 August 2023) [54], using the GOSt functional profiling to obtain the gene ontology (GO) terms for biological processes (BPs) version e109_eg56_p17_1d3191d (accessed on 30 August 2023) and for cellular component (CC) and molecular function (MF), as well as KEGG version e110_eg57_p18_4b54a898 (accessed on 28 September 2023). We use a g:SCS threshold of 0.05.

## 3. Results and Discussion

### 3.1. Differentially Expressed Genes (DEGs)

**Figure 1:**
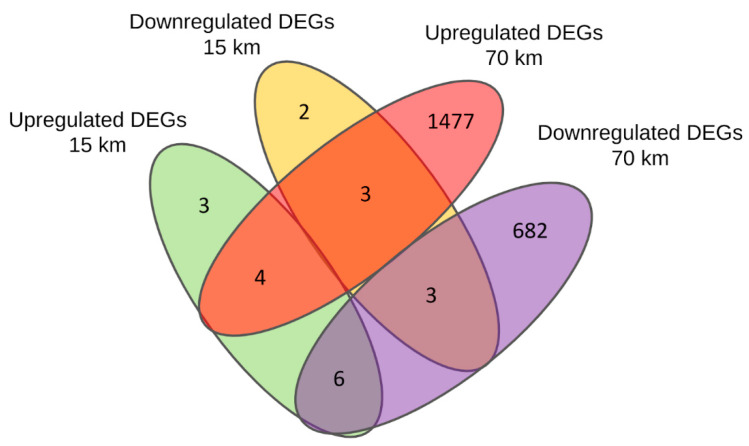
Venn diagram of the number of differentially expressed genes (DEGs) obtained for the horses pre-run and post-run that covered distances of 70 km (long-distance) and 15 km (short-distance), respectively. The diagram shows the number of upregulated and downregulated DEGs which are common or specific to the two distance groups. The 16 DEGs overlapping between long- and short-distance groups are listed in Table 1.

In the long-distance group, we found 2175 DEGs (1484 upregulated and 691 downregulated). In the short-distance group, there were only 21 DEGs (13 upregulated and 8 downregulated). The complete lists of all found DEGs in both long- and short-distance runs with gene names, fold change (FC), and false discovery rates (FDR) are given in Tables S1 and S2. The number of up- and downregulated genes that overlap between both the long- and short-distance groups can be seen in the Venn diagram (Figure 1; see also Table 1). In particular, 16 of the 21 DEGs of the short-distance group overlap with the DEGs of the long-distance group, while five apply only to the short-distance DEGs (three upregulated, two downregulated).

Since there is still a lot of nonannotated (NA) sequence information in horses, which cannot be used for the evaluation of the activated metabolic pathways, the further analyses were only carried out with the annotated genes. This means that 1275 upregulated and 580 downregulated DEGs are available for the long-distance as well as 13 up- and 5 downregulated DEGs for the short-distance group.

The large difference in the numbers of DEGs in the two distance groups is striking. The remarkably smaller number of DEGs in the short-distance group may be due to the fact that the horses were well trained and generally not ridden at maximum speed. This possibly means that the differences in gene expression between pre-run and post-run are only relatively small, because the metabolic processes have not changed much and, consequently, the individual variation between the few animals in a distance group is too large to find more significant changes. Despite the clear methodological differences in the various studies, a certain trend can be observed overall. There are usually more upregulated than downregulated genes, while the total number of DEGs is greater after heavy physical activity than after light physical activity [51]. For example, Mach et al. [25] found 2453 DEGs after a 160 km endurance ride, while our own results show 2175 DEGs after 70 km and 21 after 15 km. In contrast, after a 30 min trot, only 142 DEGs were detected in blood [17].

### 3.2. Gene Ontology Enrichment and Pathway Analysis of DEGs

The GO analysis revealed several biological processes that are associated with the endurance of the horses in long- and short-distance runs. The varying distance covered by the horses in the two runs, and thus the different endurance requirements, can give first insight into the active biological processes.

**Figure 2:**
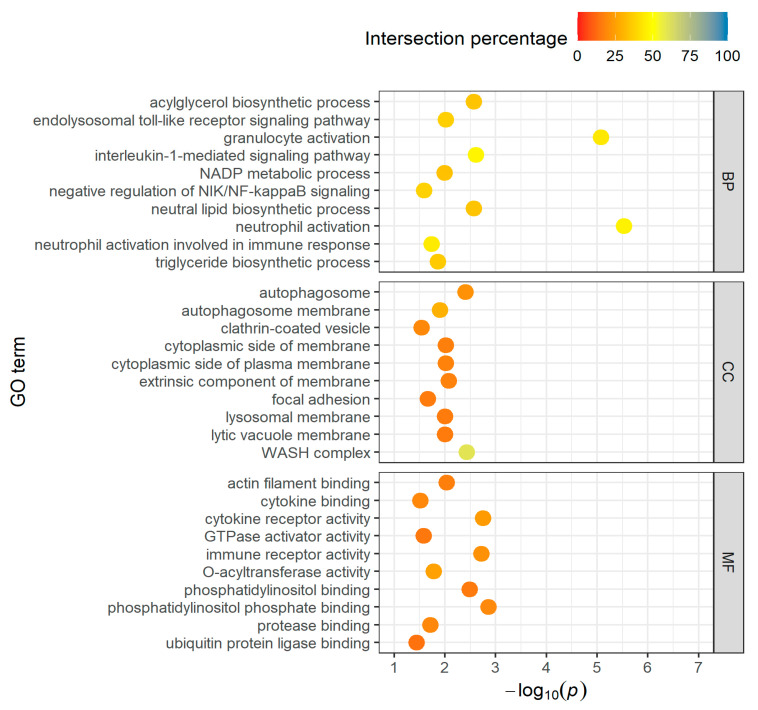
Top-10 GO terms of BPs, CCs and MFs for upregulated DEGs in the long-distance group, taking into account the proportion of found genes which are overlapping with the total number of genes in the GO term (intersection percentage) and the negative log10 of the adjusted *p*-value.

Long-distance group: Functional enrichment analysis of the 1275 upregulated DEGs representing known genes in the long-distance group shows that they are involved in 343 biological processes. In order to have a better chance of finding really important genes and metabolic processes, we focused on the processes in which the genes found account for at least 10% of the total number of genes (intersection percentage) and in which no more than 250 genes are involved in total. This constraint reduced the number of biological processes to 109, involving 457 upregulated genes (Table S3). A reduction from 43 to 15 was achieved in the cellular components (CCs) and from 40 to 10 in the molecular functions (MFs). Figure 2 show the top-10 GO terms (sorted after intersection percentage) of BPs, CCs, and MFs, respectively.

Among the top-10 biological processes (BPs), the focus is on fatty acid metabolism (acylglycerol, neutral lipid, and triglyceride biosynthetic process) and immune response (neutrophil and granulocyte activation, toll-like receptor signaling pathway). The cellular components (CCs) primarily list reactions that influence membranes, and the molecular functions (MFs) refer to enzyme reactions that are particularly related to the biological processes (O-acyltransferase and cytokine activity) mentioned but also to cell proliferation and cell membrane actions (phosphatidylinositol binding).

**Figure 3:**
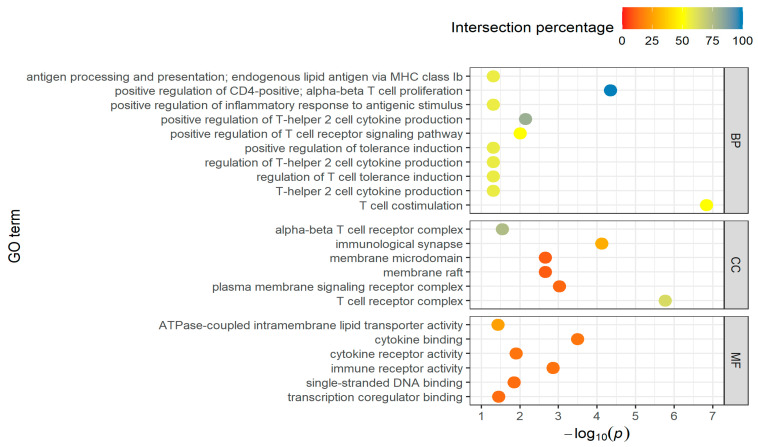
Top-10 GO terms of BPs, CCs, and MFs for downregulated DEGs in the long-distance group, taking into account the proportion of found genes that are overlapping with the total number of genes in the GO term (intersection percentage) and the negative log10 of the adjusted *p*-value.

In the downregulated DEGs of the long-distance group, the number of biological processes decreased from 209 to 104, of cellular component terms from 16 to 6, as well as of the molecular functions from 8 to 6. That results in a decrease of involved genes from 580 to 111 (Table S4). The top-10 GO BPs terms of this group are shown in Figure 3. Only six terms are available for CCs and MFs.

The identified processes are often involved in regulatory tasks and immune and stress response (T cell activation, interleukin production, leukocyte proliferation), as well as in metabolic processes (carbohydrate catabolic process, lipid biosynthesis, NADP metabolic process). The T cell receptor complex is also strongly represented in the GO:CC terms and the cytokine activity can be found under the molecular functions.

Among the top-10 upregulated genes (sorted according to FC, Table 2) involved in the important biological processes and found to be differentially expressed in our study, six genes are active in the inflammatory and the immune system (*IL22RA2*, *IL20RA*, *IL1R2*, *LFT*, *SPI2*) as well as in apoptotic processes (*CLU*). Four genes are involved in energy supply: the STBD1 protein, which is important as a cargo receptor for the transport of glycogen to the lysosomes; the *GYG1* gene, which encodes a glycosyltransferase involved in the glycogen metabolism in muscle; MOGAT1, which catalyzes the diacylglycerol synthesis; and VDR, which regulates a variety of metabolic pathways. The downregulated genes, often transcription factors, are mainly involved in immune processes. In addition to the interleukin IL5RA, the system of T and B cell differentiation and proliferation is particularly represented (*CD40LG*, *EPHB6*, *UBASH3A*, *HES1*, *FCER2*, *TRAT1*, *FOXP3*).

In numerous studies, an increased expression of various interleukins as well as an activation of T-cell-associated processes could be observed [55,56,57]. While *IL1R2* and *IL22RA2* were differentially expressed in accordance with other studies [53,58], *IL6* and *IL8* were not identified as DEGs in our study [53,58,59,60]. The strong activation of regulatory processes and immune response-related genes seems to play crucial roles in the endurance of horses [51,58,61].

The metabolic active gene *GYG1* was also found in earlier studies [16,17]. In addition to *MOGAT1*, some more genes from the lipid metabolism, such as *PLIN5* (FC = 5.66) or *SIK1* (FC = 3.5), were identified as DEGs, albeit not in the top-20 group.

Short-distance group: In the short-distance group, only few DEGs could be found (see Table S2). The 13 upregulated DEGs take part in such different processes that no significant ontology enrichment analysis was possible. Some genes could only be assigned to interesting biological processes. For example, *FOS*, *FOSB*, and *PRF1* play a role in “cellular response to calcium ion” (GO:0071277). *CCL5*, *FASLG*, and *TRAT1* are involved in the “positive regulation of phosphatidylserine exposure on apoptotic cell surface” (GO:1905782). *IGF2* acts in the “glucose metabolic process” (GO:0006006) while *PRF1* is involved in the “Type I diabetes mellitus signaling” pathway.

**Figure 4:**
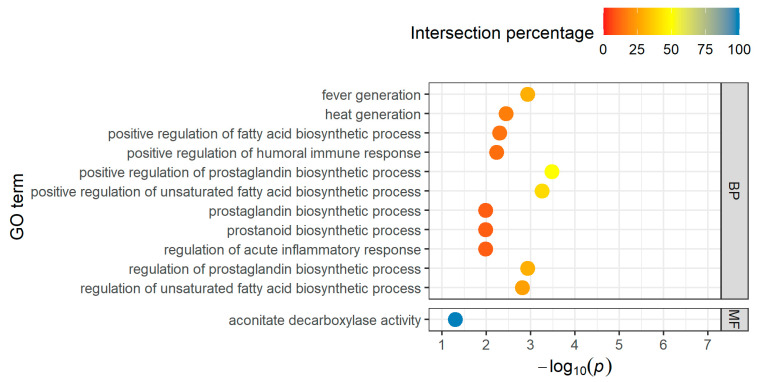
Top-11 BP terms and one MF term for downregulated DEGs in the short-distance group, taking into account the proportion of found genes that overlapped with the total number of genes in the GO term (intersection percentage) and the negative log10 of the adjusted *p*-value.

The five annotated downregulated genes are integrated in 32 biological processes, no cellular component, and one molecular function term (Table S5). After filtering using the intersection percentage, eleven biological processes and one molecular function GO term with four genes each appear to be particularly relevant (Figure 4).

The two main genes, *IL1B* and *PTGS2*, are important for the “regulation of prostaglandin biosynthetic process” (GO:0031392, GO:0031394) and the “regulation of fatty acid biosynthetic process” (GO:0045723, GO:2001279, GO:2001280). For the immune and inflammatory response, which also plays a role in this group, *ACOD1* and *NLRP3* are also involved. *ACOD1* is the only gene which acts in the molecular term “aconitate decarboxylase activity”.

Despite the considerably lower number of DEGs compared to the long-distance group, regulatory processes come first. The strong involvement of the response to calcium ion is interesting and can be a reaction to oxidative stress within the blood during the endurance run. When examining Standardbred racehorses with recurrent exertional rhabdomyolysis, calcium ion regulation was significantly downregulated [37]. The activation of the Ca^2+^-sensitive K^+^ channel increases the K^+^ efflux and results in shrinkage of the cell and breakdown of phosphatidylserine asymmetry. It also increases the flow of Ca^2+^ within the cells and further makes phosphatidylserine translocate to the membrane of erythrocytes, thus triggering the death of erythrocytes [62].

This assumption is supported by the upregulation of *FOS* in muscle found in a test leading to maximum heartrate immediately [16]. Interestingly, the GO category “regulation of prostaglandin biosynthetic process” is relatively well represented here. Genes in this group are involved in blood flow, anti-inflammatory effect, and pain perception [63,64]. The importance of prostaglandin and proinflammatory cytokines (e.g., IL8B) in connection with endurance was shown in a stress test of Thoroughbred horses, where, among other things, a significant increase in prostaglandin plasma concentration was found [60].

**Figure 5:**
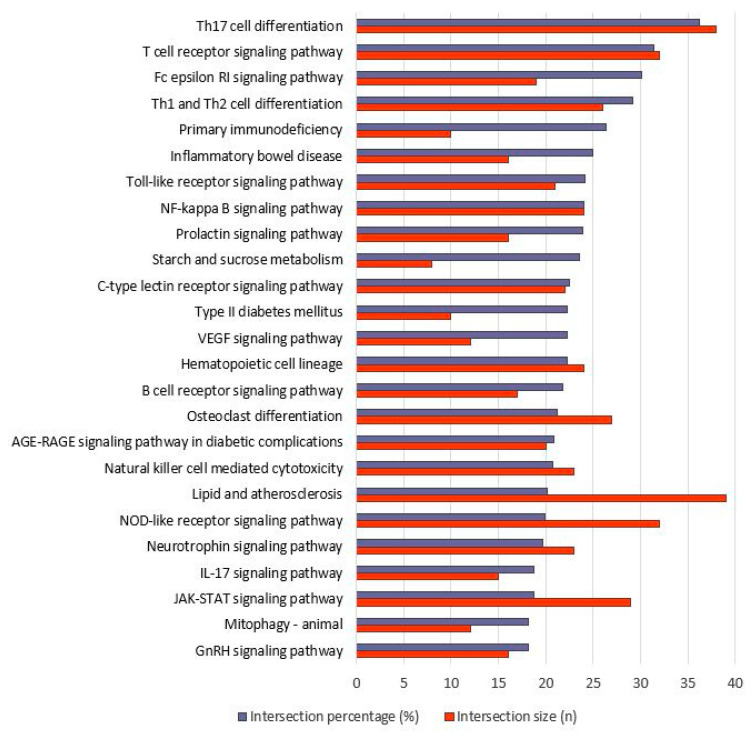
Top-20 KEGG pathways of up- as well as downregulated genes after long-distance run (sorted after intersection percentage). KEGG categories are shown along the *Y*-axis, while both intersection percentage (proportion of intersection size to term size in %) and intersection size (number of genes that overlapped between KEGG category genes and found DEGs) are shown along the *X*-axis.

Pathway analysis: Using KEGG analysis, we obtained 81 significantly overrepresented pathways in the long-distance group. Of these, 48 pathways are of particular interest because 8 are primarily associated with cancer and 25 with infectious diseases (Table S6). The top-20 (sorted according to intersection percentage) are shown in Figure 5.

Upon closer inspection, it can be seen that pathways that are integrated into both the immune response and the anti-inflammatory system are significantly overrepresented. This group includes T, T helper, and B cell cascades which are playing an important role in the adaptive immune system. A total of 38 of the genes we found are integrated into the “Th17 cell differentiation” cascade and 32 genes are involved in the “T cell receptor signaling pathway”, each of which accounts for around a third of all genes contained in this pathway. Another pathway is the “Fc epsilon RI signaling” cascade, which regulates the release of inflammatory mediators such as cytokines, interleukins, and prostaglandins. The importance of the genes in this complex in coping with stress when faced with high demands has already been demonstrated in various studies both at the molecular and protein levels. For example, well-trained horses showed an anti-inflammatory reaction after an 800 m race, while untrained horses showed a proinflammatory reaction, which is considered a training effect [42,65]. After an appropriate period of rest, these changes appear to return to normal, at least partially [44]. IL1RA, which correlates with cortisol concentration, appears to be of particular importance in restoring the homeostatic equilibrium [45]. In our studies, we also found some of these genes, for example, *CD4* (FC = −1.74) or *FOXP3* (FC = −2.44), to be differentially expressed as well as the overrepresented “IL-17 pathway”.

The top-25 pathways also include the “toll-like receptor (TLR) signaling” and the “NF-kappa B signaling” cascade. TLRs induce kinases and pathways including IL6 (chemokine), which results in the expression of inflammation- and immune-response-related genes [58]. In our study, the *MYD88* gene was significantly upregulated (FC = 2.15). This gene encodes an adaptor protein which acts as a mediator of the NF-kappa B signaling starting from toll-like receptors until the nucleus, finally resulting in the expression of interferon, and inflammation-related cytokine genes [66]. As a consequence, inflammation occurs as a stress response, which in turn could be activating pathways for maintaining homeostasis. This could be the major reason for the overrepresentation of several immune-response-related pathways, especially in the long-distance group. Platelet activation and aggregation could be occurring as a result of the activation of inflammation-related pathways, particularly for tissue repair following physiological adaptation and homeostasis [67]. The “hematopoietic cell lineage pathway”, in which 24 of our DEGs are involved, also works in this direction. “TLR signaling” and “NF-kappa B signaling” as well as “NOD-like receptor signaling” (cooperating with TLR in inflammatory and apoptotic response) and “JAK-STAT signaling” (playing a crucial role in myogenesis) pathways were found as overrepresented and to be involved in stress response after exercises in various studies [68,69,70].

For the downregulated genes of the short-distance group, only five pathways are overrepresented. Three are connected with “C-type lectin receptor signaling”, “IL-17 signaling”, and “cytosolic DNA-sensing” pathways, while the first two are also found in the top-25 pathways of the long-distance group. Two further pathways are strongly associated with infectious diseases (Table S7).

The significantly changed expression in several genes and the reflection in the pathway activation reflects the metabolic changes of an organism as a reaction to the demands of an endurance run. Some genes are characterized by a high reproducibility of both their induced expression and changed serum level of the proteins. This group includes the interleukins IL1B, IL1R2, and IL22RA2, whose genes were also expressed differentially in our study, as well as IL6, IL8, IL13, and IL17, which were found in other expression or enzyme studies [42,43,44,45,46,55,56,60,65,71]. Various studies on other serum values are interesting in this context. While, previously, the lactate concentration [72,73] was primarily used as a parameter for the degree of exhaustion, today, hormones such as testosterone and cortisol are also used for evaluation in addition to cytokines. An anabolic index determined from the ratio of these hormones is a possible parameter but is more suitable for racing horses than for endurance horses [74]. Differences in expression of these genes could therefore possibly indicate individually varying adaptability and could thus be markers for the monitoring to avoid training overload as well as for identifying horses with higher stress resistance [68]. Although there are already numerous studies in this area that determine gene expression or enzyme serum levels, there is still a lack of data and reference values, especially in the combination of such datasets. In addition to the individual variation in these values, this is primarily due to the extremely different test conditions (trained vs. untrained horses, training effect over a certain period of time, short races vs. long and very long endurance rides, different breeds, and sick vs. healthy horses [21,40,43,46,75]. In the future, determining the expression of these genes or the concentration of their proteins in the serum could provide information about the adaptation of individuals to the requirements of performance challenges and thus be markers for stress management. This would enable the training and sporting use of horses to be optimized as well as monitored and overtraining syndrome (OTS) to be avoided [40,51,57,76,77]. This would be a big step towards improving welfare. Whether the horse can be a model for humans in the field of endurance [46,65] should be discussed, as the horse’s physiology shows considerable differences in how it copes with heavy muscle strain and oxygen supply.

### 3.3. Search for Candidate Genes for Endurance Performance

The analyses of our data so far show that genes and biological processes that are associated with cellular regulation, apoptosis, or immune, as well as inflammatory, response are subject to strong activation, regardless of the specific exercise type, playing an important role in coping with stress. In addition to this group, genes are also of interest, which can be candidates for performance selection for endurance based on their function in the metabolism. Therefore, we focused our attention on 16 DEGs which are represented in both distance groups (see Table 1). Two of these (upregulated in long-distance and downregulated in short-distance runs), which have no annotation will not be considered in detail.

**Figure 6:**
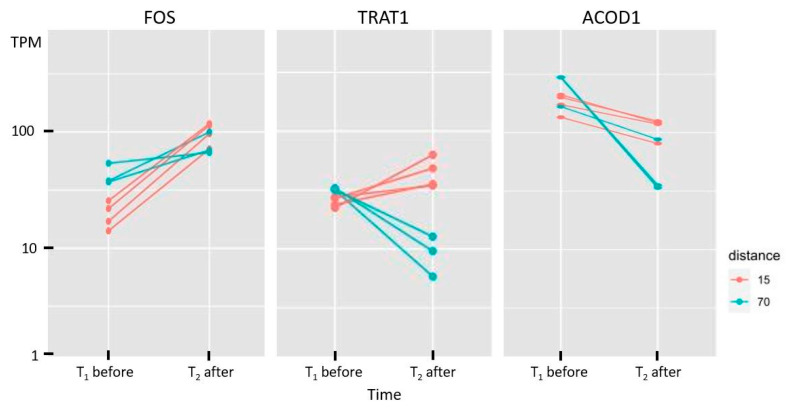
Comparison of the expression level (in transcripts per million, TPM) before and after the run in three DEGs that are congruent (*FOS*: up–up, *ACOD1*: down–down) or opposite (*TRAT1*: down–up) in both distance groups (long: 70 km, short: 15 km).

An example of the changes in expression of three genes in terms of transcripts per million (TPM) before and after the run is given in Figure 6. The expression changes in all genes in both groups (long- and short-distance) are given in Figure S1.

However, a look at the function of these genes shows that most of them are also associated with inflammatory processes and stress response. The already-mentioned genes *FOS* and *FOSB*, upregulated for long- and short-distance runs, are AP-1 transcription factor subunits which play a major role in regulating the osteoblast differentiation at the early stages and are known for their role in phospholipid synthesis [78]. An upregulation of *FOS* was also found in previous analyses in both muscle and blood [16,17]. This could be the reason for the crucial role of *FOS* in adaptation of the animal after an endurance run. Both genes are also involved in processes of lipodystrophy. But in context with endurance, their function in cell death activation of T cells by binding on the *FASLG* promotor, IL-17 pathway, and apoptosis cannot be underestimated and may be the main reason for their differential expression. *LMNA*, which is extensively involved in the maintenance of skeletal muscle strength and osteoblastogenesis, as well as in apoptosis, and *NLRP12*, acting as regulator of inflammatory response, are also upregulated genes for both distances. An interesting group includes genes with opposite changes in the two distance groups. *NLRP3*, a paralog of *NLRP12*, is one of the genes for which expression levels increased in animals that performed the long-distance run, whereas they decreased in animals during a short-distance run. It plays a major role in the formation of the cytosolic multiprotein complex called NLRP3 inflammasome, and is therefore involved in the NF-kappa B signaling and the regulation of innate immune responses, cell death, and inflammation [71,79,80]. This group also comprises the *FASLG* gene, a member of the tumor necrosis factor superfamily which is important for the regulation of the immune system [81], *TRAT1*, part of the T cell receptor complex, and the chemokine gene *CCL5*, which has been reported as a candidate gene in previous studies [51,53]. The opposite direction of regulation, also seen in *ADGRG1* (inhibitor of tumor progression) and *RETREG1* (acting in neuro survival), can point to a lack of available energy in the long-distance run when all nonessential biological processes are shut down as much as possible. It is remarkable to note the downregulation in the long-distance group of *PSD3*, which reduces the intracellular lipid content of the liver [82], and which is therefore integrated into the process of energy supply. The genes *ACOD1*, *IFIT2*, and *PTGS2*, which are downregulated in both distances, are involved in processes of apoptosis and in the inflammatory system.

In the search for candidate genes, Park et al. [17] compared their expression profiles with known racing-performance-related genes [83] and found, among other things, *HIF1A* to be upregulated. This transcription factor subunit is involved in energy metabolism, which is increasing oxygen delivery and metabolic adaptation to hypoxia. In our analysis, *HIF1A* is not in the top 10, but is also significantly upregulated (FC = 2.33). This is also in agreement with the results of a study in Thoroughbred horses [84]. Furthermore, *HIF1A* can stimulate promotor elements of *PFKFB3* [85], a regulatory molecule that controls glycolysis, which is upregulated (FC = 9.14) in our study but also in other studies [16]. Changes in the glucose as well as the insulin pathway were repeatedly pointed out [26,29], which we can confirm based on specific DEGs (*MOGAT1*: FC = 16.01; *GYG1*: FC = 12.45) and pathways (“Type II diabetes mellitus”) that we have identified.

The DEG *PPARD* involved in the “PPAR signaling pathway” and found as a significant DEG in many studies [76,86,87] did not show significantly changed expression in our analysis. Only *PPARG* was differentially expressed. Of the other genes influencing potential racing performance [83], *GYG1* and *VDR*, which is a transcription factor and mainly involved in mineral metabolism, were upregulated. In blood, the transcription factor GATA2, which is important for development and proliferation of hematopoietic stem cells, was downregulated (FC = −5.49), which was also reported in previous studies [17,53]. Above all, the provision of energy is important for a horse’s performance. Of course, it must be taken into account that when switching from short-term exercise to endurance exercise, the horse also switches from the anaerobic to the aerobic metabolism [34]. For humans, there is already a review that includes DNA polymorphisms associated with athletic performance. There are already 41 endurance-related, 45 power-related, and 42 strength-related genetic biomarkers [88]. Such a gene and SNP collection could be a model for research on horses.

In order to achieve an even better understanding about the importance of the various DEGs for endurance performance and to foster a more targeted selection of the genes to be examined in more detail, the results of expression studies should be combined with GWAS analyses [89], investigations of transcription regulation (e.g., miRNAs or methylation analysis [25,29,40]), and SNP identification. Last, but not least, these findings should be compared with the response in the blood and muscles [90].

## 4. Conclusions

In our study, mainly DEGs from the area of general regulation and both the immune and stress response were found after endurance runs in horses. These genes show a fast response in blood and could therefore be candidates for quick and easy monitoring of stress of horses in training as well as in competition, improving animal welfare and avoiding the overtraining syndrome and overload in runs. On the other hand, there is interest in genes that could be responsible for individual endurance disposition. It would be promising to look for mutations in the genes themselves as well as in their regulatory regions. These specific candidate genes might serve as potential markers for selection in future breeding programs.

## Figures and Tables

**Table 1:** DEGs which were found in both long- and short-distance runs.

Gene Name	Long-Distance Run		Short-Distance Run	
	FC ^(1)^	Class	FC ^(1)^	Class
*FOS*	2.24	up	4.92	up
*FOSB*	3.18	up	9.00	up
*LMNA*	3.05	up	2.62	up
*NLRP12*	2.60	up	1.76	up
*NLRP3*	1.72	up	−1.52	down
*ADGRG1*	−1.95	down	1.70	up
*CCL5*	−1.82	down	1.75	up
*FASLG*	−2.05	down	1.62	up
*PSD3*	−1.59	down	1.50	up
*RETREG1*	−1.84	down	1.53	up
*TRAT1*	−2.47	down	1.51	up
*ACOD1*	−2.17	down	−1.59	down
*IFIT2*	−2.20	down	−1.53	down
*PTGS2*	−3.28	down	−1.76	down

^(1)^ Fold change (log2FC).

**Table 2:** Top-10 up- as well as downregulated DEGs (sorted according to FC) that are involved in the selected GO terms (after filtering according to intersection percentage).

Ensembl ID	Gene Name	FC ^(1)^	FDR ^(2)^	Number of GO Terms ^(3)^		
				BP	CC	MF
ENSECAG00000008817	*IL22RA2*	143.30	0.000	3	0	3
ENSECAG00000007642	*IL20RA*	57.45	0.000	2	0	1
ENSECAG00000000288	*IL1R2*	40.73	0.000	2	0	3
ENSECAG00000029043	*STBD1*	20.45	0.000	10	0	0
ENSECAG00000021499	*LTF*	18.01	0.000	16	1	0
ENSECAG00000015822	*VDR*	16.68	0.000	2	1	0
ENSECAG00000005838	*MOGAT1*	16.01	0.000	5	0	1
ENSECAG00000007010	*CLU*	15.46	0.000	13	0	1
ENSECAG00000014414	*SPI2*	15.11	0.000	1	0	1
ENSECAG00000023192	*GYG1*	12.44	0.000	4	0	0
ENSECAG00000022503	*IL5RA*	−4.80	0.000	3	0	3
ENSECAG00000011392	*CD40LG*	−3.33	0.000	30	0	0
ENSECAG00000006853	*UBD*	−3.28	0.000	2	0	0
ENSECAG00000007276	*EPHB6*	−2.86	0.000	16	0	0
ENSECAG00000032819	*UBASH3A*	−2.81	0.000	6	0	0
ENSECAG00000026945	*HES1*	−2.60	0.002	19	0	0
ENSECAG00000017615	*CDH17*	−2.53	0.015	4	0	0
ENSECAG00000009646	*FCER2*	−2.52	0.000	12	0	1
ENSECAG00000021248	*TRAT1*	−2.47	0.000	8	2	0
ENSECAG00000022667	*FOXP3*	−2.44	0.003	79	0	0

^(1)^ Fold change (log2FC); ^(2)^ False discovery rate; ^(3)^ BP: biological process; CC: cellular component; MF: molecular function.

## Data Availability

The data will be made available after acceptance of the publication.

## Supplementary Materials

The following supporting information can be downloaded at: https://www.mdpi.com/article/10.3390/genes14111982/s1, Figure S1: Gene expression in terms of transcripts per million (TPM) of genes which are significantly differentially expressed in both short- and long-distance runs; Table S1: List of all DEGs for the long-distance run with FC ≥ 1.5 as well as FC ≤ −1.5; Table S2: List of all DEGs for the short-distance run with FC ≥ 1.5 as well as FC ≤ −1.5; Table S3: Gene ontology (GO) enrichment analysis for upregulated genes involved in the long-distance run; Table S4: Gene ontology (GO) enrichment analysis for downregulated genes involved in the long-distance run; Table S5: Gene ontology (GO) enrichment analysis for downregulated genes involved in the short-distance run; Table S6: KEGG pathway analysis for up- and downregulated genes found in the long-distance run; Table S7: KEGG pathway analysis for downregulated genes found in the short-distance run.
