# Supplementary figures and images for: Identification of Differentially Expressed Genes after Endurance Runs in Karbadian Horses to Determine Candidates for Stress Indicators and Performance Capability
Source: Genes (Basel). 2023 Oct 24;14(11):1982. doi: 10.3390/genes14111982 (PMC10671444; doi:10.3390/genes14111982)

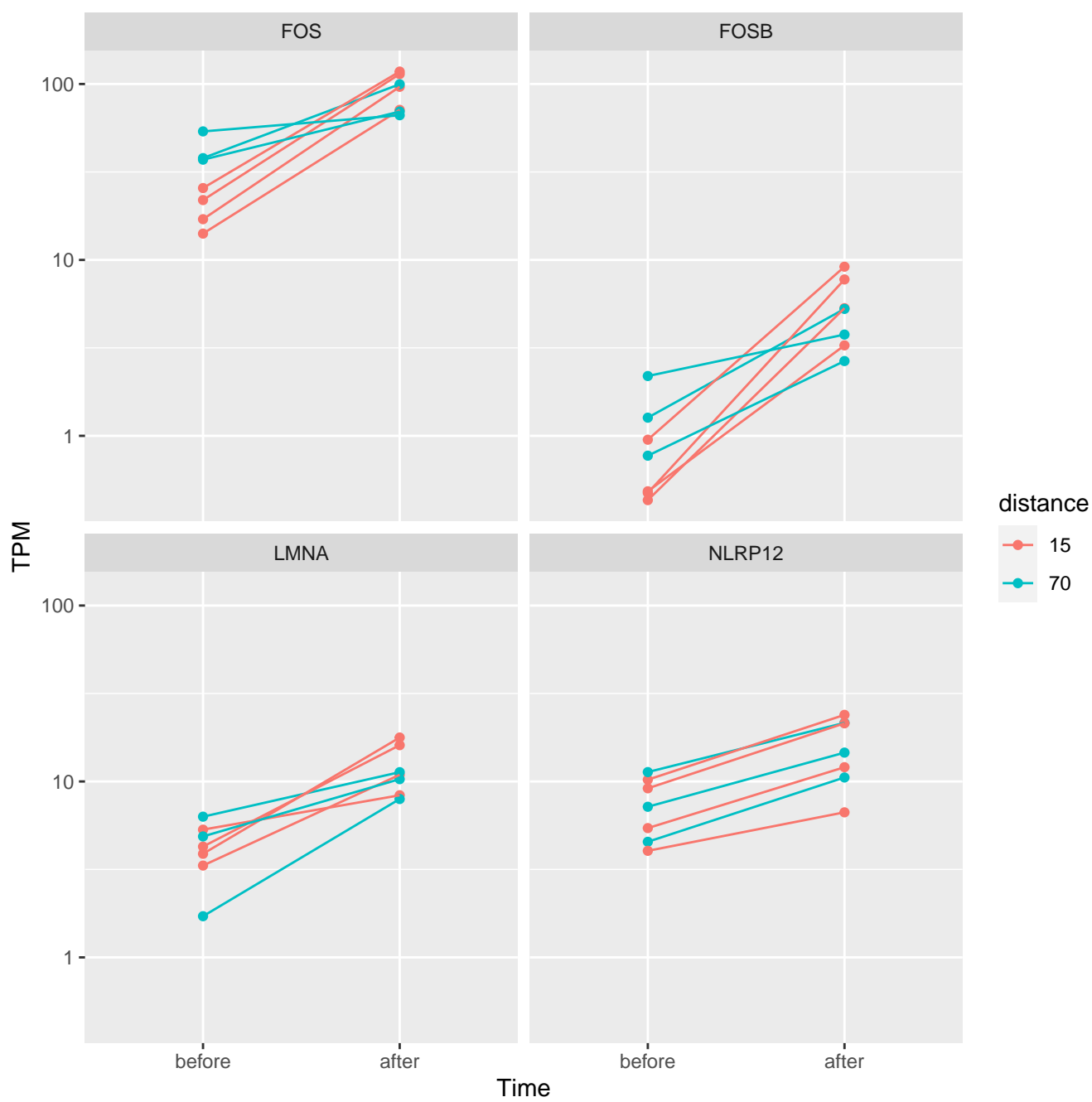

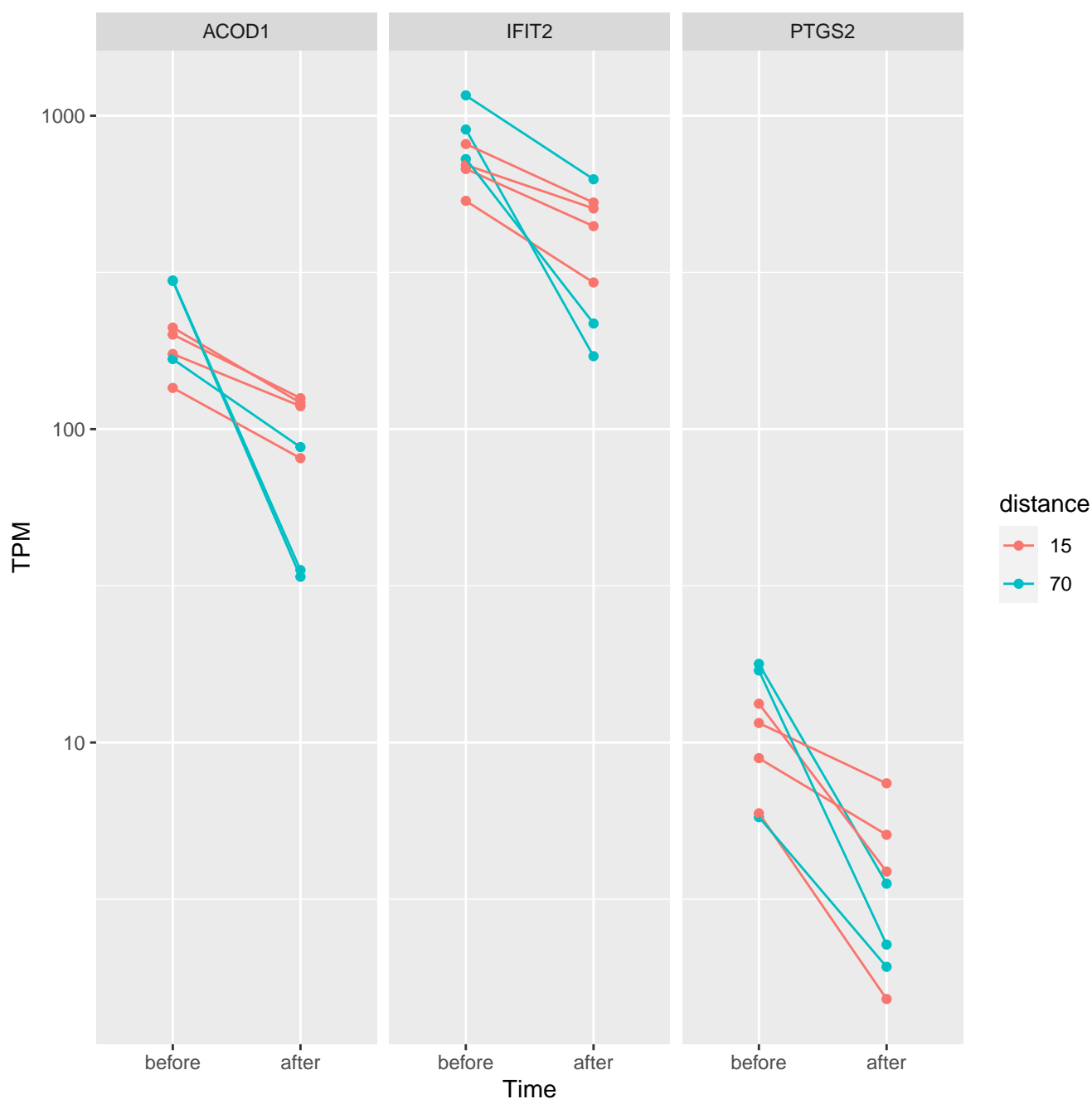

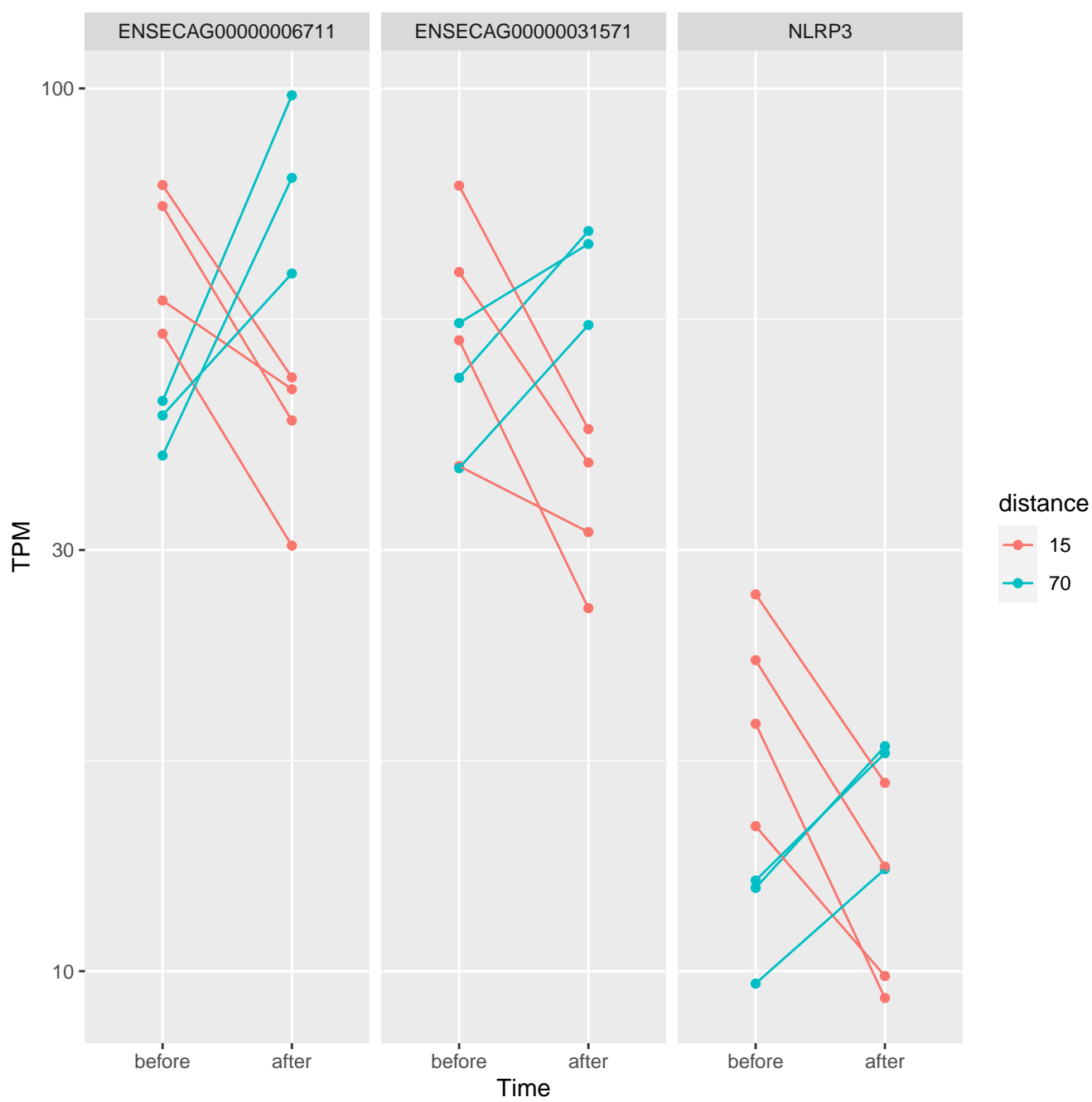

TPM

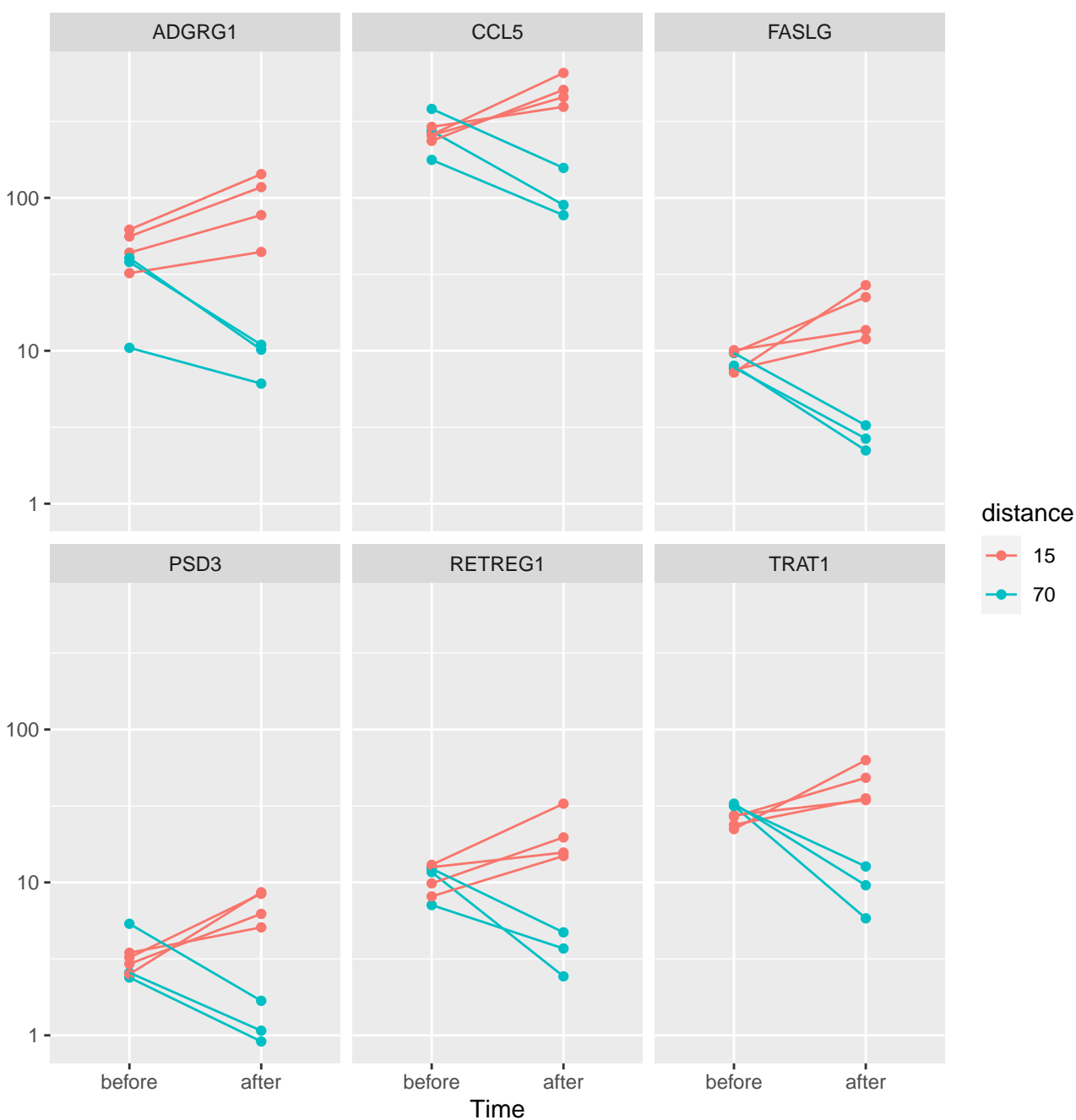

Supplement: Supplementary file 1 [file genes-14-01982-s001.zip › Figure S1.pdf]
